# Application of multiplet structure deconvolution to extract scalar coupling constants from 1D nuclear magnetic resonance spectra

**DOI:** 10.5194/mr-2-545-2021

**Published:** 2021-07-06

**Authors:** Damien Jeannerat, Carlos Cobas

**Affiliations:** 1 NMRprocess.ch, Geneva, 1200, Switzerland; 2 Mestrelab Research S.L. Santiago de Compostela, A Coruña, 15706, Spain

## Abstract

Multiplet structure deconvolution provides a robust method to determine the values of the coupling constants in first-order 1D nuclear magnetic resonance (NMR) spectra. Functions simplifying the coupling structure for partners with spin larger than 1/2 and for doublets with unequal amplitudes were introduced. The chemical shifts of the coupling partners causing mild second-order effects can, in favourable cases, be calculated from the slopes measured in doublet structures. Illustrations demonstrate that deconvolution can straightforwardly analyse multiplet posing difficulties to humans and, in some cases, extract coupling constants from unresolved multiplets.

## 1 Introduction

When studying organic compounds, 1D ${}^{1}H$ nuclear magnetic resonance (NMR) spectra are often the only, and sufficient, analytical method engaged. All chemists know how to describe simple multiplet structures. It consists of determining their position (chemical shift), integral and, when possible, coupling structure by identifying doublets ( d ), double doublets ( dd ), etc. Any serious analysis also includes the values of the scalar coupling constants. Indeed, the multiplet structures provide important topological and structural information on organic compounds and natural products. For example, the presence of a methyl group on a carbon bearing a proton will produce a quartet structure, the conformation of a double bond has clearly distinct geminal coupling constants, dihedral angle influences vicinal coupling constants (Karplus, 1963), etc.

These NMR parameters are not only helping researchers to identify their products, but they also allow reviewers to assess the validity of the argument supporting their identification and benefit the community by providing very precious information when structurally similar compounds are encountered. It is therefore of fundamental importance to provide chemists with the most powerful tools to analyse multiplets, in particular in the cases where the multiplet structure is too complex to be deciphered by visual inspection because of high degeneracy (complex structures such as “*dqd*”), partial overlap of multiplet structures or second-order effects.

The measurement of scalar coupling constants has been the subject of very intense academic work, either for direct application to 1D spectra (McIntyre and Freeman, 1992; del Río Portilla and Freeman, 1993) or involving the development of NMR pulse sequences producing spectra where coupling constants can be measured easily (Marquez et al., 2001), even in situations of extensive overlap (Prasch et al., 1998; Kiraly et al., 2020; Berger, 2018). The sad observation is that the impact of these developments is extremely limited because the broad community of chemists almost exclusively relies on basic 1D ${}^{1}H$ spectra to extract coupling constants. Even if users of NMR were aware of them, they would be reluctant to use experiments going against the common practice in their fields except if they were producing key results – something NMR coupling constants alone rarely do.

We shall report here on a significant improvement of the analysis of 1D ${}^{1}H$ spectra based on multiplet-structure deconvolution which will be introduced in a future release of Mestrelab's Mnova software (Mnova NMR version 14.3.0, 2021). This method was originally developed during the 90s to automatize the analysis of multiplet structure from spectra generated by one of the forgotten pulse sequences: the soft-COSY experiment (Emsley et al., 1990). Multiplet deconvolution proved to be applicable to the more “standard” DQF-COSY experiment (Jeannerat, 2000) after simply increasing the direct acquisition time to provide higher resolution – something which was problematic because of the data storage available at that time but which is no longer justifiable. We shall demonstrate the power of the application of multiplet deconvolution outside the world of 2D correlation spectroscopy, where positive and negative peaks coexist and cause specific challenges (Jeannerat and Bodenhausen, 1999), and apply it to the common – not to say mundane – multiplet structure present in standard 1D NMR spectra.

### 1.1 Multiplet structure in 1D spectra

**Figure 1:**
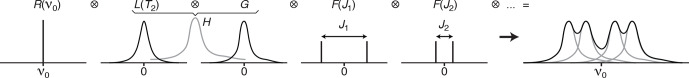
Multiplet structure of a 1D NMR spectrum expressed as the convolution product of the contributions of the Larmor frequency of the nucleus $R(\upsilon_{0})$ , the Lorentzian line shape $L(T_{2})$ , the $B_{0}$ -field inhomogeneity G(inhom.) and the scalar coupling constants $F(J_{i})$ .

For weakly coupled spin systems, the multiplet structure observed in 1D spectra can be seen as the result of the combination (see Fig. 1) of the resonance frequency, the effects of the exponential relaxation, $B_{0}$ -field inhomogeneity and scalar coupling ( J ) interactions (Metz et al., 2000).

In the frequency domain, the ⊗ symbol stands for the “convolution product” which corresponds, in the time domain, to pointwise multiplication. The detected FID is, indeed, the product of the chemical shift evolution, the exponential decay (producing a Lorentzian shape L(υ) ), a function reflecting the effect of $B_{0}$ inhomogeneity (often modelled as Gaussian shapes in both domains) and cosine modulations caused by the first-order J -coupling interactions (resulting in a doublet in the 1D spectrum).

In liquid-state NMR and in isotropic media a doublet F(J) is expressed, mathematically, by a pair of so-called δ functions, i.e. a function returning zero values everywhere except for the values of v=-J/2 and +J/2 , where J is the scalar coupling constant. When taking the convolution product of any set of δ functions with any other function, typically a spectral line shape H , the position and amplitude of the δ functions can be understood as indicating where the convoluted function H should be duplicated before summation (see the grey components in the right part of Fig. 1).

The deconvolution consists in reversing the effect of the convolution of a given function. The symbol ⊘F is sometimes used to represent the process reversing the effect of ⊗F . However, what seems to be a simple division is misleading because, in reality, the ⊗ symbol stands for an integral which has no general inverse. One should, instead, look for an inverse of the F function and replace the deconvolution operator with the convolution with an inverse function ⊗1/F(J) . An alternative is to operate in the time domain, but the simplicity of the division is compensated for by the difficulty in dealing with division by zero.

Whether it is applied in the time or frequency domain, deconvolution is a difficult process prone to producing noise and artifacts. The low efficiency of image deblurring is a reminder of this fundamental difficulty. In the field of NMR, methods improving the spectral line shape (relaxation and field inhomogeneity) were developed in the group of Gareth A. Morris under the general framework known as *reference deconvolution* (Morris et al., 1997; Morris, 2002), but the difficulty in automating them makes deconvolution disappointingly disregarded. We shall not discuss, here, the deconvolution of spectral line shapes but shall concentrate on the deconvolution of the effect of coupling interactions called “multiplet-structure deconvolution”. Following developments in the time domain (Le Parco et al., 1992; Bothner-By and Dadok, 1987; Prost et al., 2006), the frequency domain became more popular to avoid the back-and-forth Fourier transformation. This branch of research started in the 1980s in the group of R. Freeman (del Río Portilla et al., 1994), continued in the 1990s in Geoffrey Bodenhausen's group (Huber and Bodenhausen, 1993a, b; Jeannerat, 2000; Jeannerat and Bodenhausen, 1999) and continued to resonate when the ACCA method (Cobas et al., 2005) was developed for Mestrelab's software Mnova.

This paper presents a follow-up of one published in 1999 (Jeannerat and Bodenhausen, 1999) which showed how to effectively use a set of inverse functions of F to obtain, through a recursive procedure (Novič and Bodenhausen, 2002), the list of the coupling constants and the line shape G . The inverse function $M(J^{*})$ , reminiscent of the set of δ functions seen in the 1990s (del Río Portilla et al., 1994), is discussed in the next paragraph. While earlier work focused on the simplification of a simple doublet, we will present applications to the deconvolution of structures originating from the coupling with partners with S>1/2 and deal with partially overlapping multiplet structures. It is generally limited to the analysis of first-order multiplets, but a method to accommodate some “roof effects” is discussed (i.e. deal with the unequal amplitude of the two lines of doublets caused by second-order effects; see Sect. 1.7) and turns it into an advantage to determine, in favourable cases, the chemical shifts of the coupling partners.

### 1.2 Deconvolution of doublet structures

**Figure 2:**
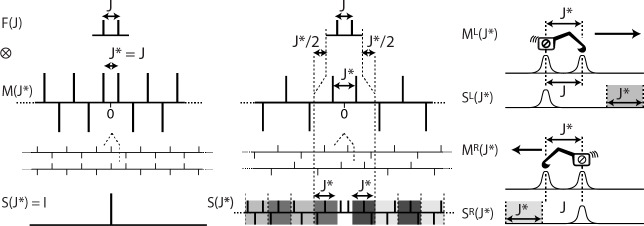
(Left) The function $M(J^{*})$ cancels the doublet of a weakly coupled partner with a spin 1/2 when $J^{*}=J$ . (Middle left) Duplicating M and shifting one copy by the distance J indeed cancels all except the central peak, resulting in the expected simplification (bottom left). (Centre) When $J^{*}\neq J$ , the convolution product results in the complex set of δ functions S (middle bottom). (Right) Illustration of the algorithm starting at one side of the spectrum. It can be seen as walking from one boundary of the multiplet, in the manner of an integrator, and subtracting, at the distance $J^{*}$ , the amplitude measure locally.

The process we shall use to reverse the effect of a doublet is the convolution product of the starting multiplet with the simplification function $M(J^{*})$ (see Fig. 2). The deconvoluted function will usually be an experimental multiplet with a possibly complex structure, but the top of Fig. 2 shows how it applies to the model function of a doublet $F^{1/2}(J)$ to provide the mathematical demonstration that M simplifies any doublet structure. The function $M(J^{*})$ is a doubly infinite series of δ functions separated by $J^{*}$ . Their signs are positive for the pair located at $\pm J^{*}$ and alternate signs when running away from this core along the two directions of the abscissa.

The simplification of the doublet structure into the singlet I is obtained when the distance between the δ functions of $M(J^{*})$ corresponds to the true coupling (i.e. when $J^{*}=J$ , bottom left in Fig. 2). Otherwise $S=F(J)⊗M(J^{*})$ consists of a complex set of δ functions that we call deconvolution artifacts (bottom centre in Fig. 2). Determining the coupling constants consists in measuring the degree of simplification of the result S as a function of $J^{*}$ . In principle, S should be infinite arrays, but beyond the boundaries of the experimental multiplet structures (see the section framed by dotted lines in the centre of Fig. 2) two patterns (in four shades of grey) repeat themselves with alternating signs, rendering the useful part of the S series finite.

In the perspective of speeding up calculations, it has been demonstrated (Jeannerat, 1997; Aksel Bothner-By​​​​​​​, personal communication, 1995) that the result of the application of the arrays of δ functions $M(J^{*})$ is equivalent to a recursive process starting on either side of the multiplet illustrated in the right part of Fig. 2. It shows that the calculation can be understood as the result of moving a cursor (represented by the excavator) over the vector of signal amplitudes and subtracting, at arm's distance, representing the tested value of $J^{*}$ , the value measured at its current position. After chopping off the margins which should be empty (in grey) to align the sub-multiplets, the sum of $S^{L}$ and $S^{R}$ is numerically equal to S . This approach has the advantage of being computationally quite effective: each point in the deconvoluted spectrum is calculated as the sum of only two points instead of $\left[W/J^{*}\right]$ , where W is the width of the multiplet region. When $J^{*}=J$ , the remote subtraction eliminates the second occurrence of the substructure split by J . A key property of this algorithm is that artifacts and the residuals of sub-multiplet subtraction increase from the starting boundary to the other as the number of operations increases. It is tempting to reduce artifacts by taking only the most favourable half of the simplified multiplets, but it is not generally recommended because it often produces a small discontinuity in the middle of the resulting multiplet. The most significant advantage of the side-to-side process is to facilitate the measure of the similarity of $S^{L}$ and $S^{R}$ to test the success of the simplification process. It takes advantage of the presence of deconvolution artifacts, indicating that $J^{*}\neq J$ only in the right or left marginal regions (in grey in Fig. 2) of $S^{L}$ and $S^{R}$ . Instead of the classical Chi-squared test, we favoured a scalar product (Huber and Bodenhausen, 1993a) as a measure of similarity, but this should only be a matter of personal preference. Finally, the presence of two sub-multiplets makes it possible to simply discard one of them when an artifact (solvent singlet, spike, etc.) or partial overlap makes the complementarity fail (see Sect. 1.8).

### 1.3 Recursive simplification of multiplet structure

**Figure 3:**
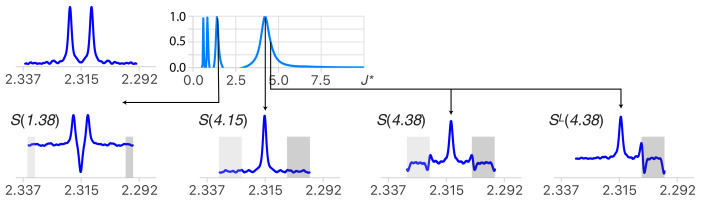
(Top right) Quality function of the deconvolution of an experimental doublet (top left). When starting with large values of $J^{*}$ , the first extremum points to the largest coupling constant of the multiplet structure ( $J^{*}$ = 4.15 Hz ). (Bottom) Structures obtained for deconvolution with $J^{*}$ = 1.38 ( =J/3 ), 4.15 ( =J ) and 4.38 ( =J+0.23 ) Hz and $M^{L}$ for 4.38 Hz . When $J^{*}\neq J$ , the presence of deconvolution artifacts (in the grey area) breaks the symmetry of the structure and causes a drop in the quality function except when $J^{*}$ = J/(2n+1) , where $n\in N^{*}$ . (See text for more details.)

As mentioned earlier, deconvolution produces artifacts because the components of multiplets are never perfectly identical. This causes imperfect multiplet subtraction due to the presence of random noise, etc. These artifacts tend to be duplicated for each δ function of M , and their presence is often a limiting factor for the analysis of complex structures. Reducing them is therefore decisive for making multiplet deconvolution reliable. Deconvolution with large values of $J^{*}$ generates fewer artifacts because they contain less δ function per unit distance. This is one of the reasons to aim first at the largest coupling present in any multiplet. Note that it is the opposite to what a human would do based on the knowledge that only the smallest coupling can be readily measured between the first and second outermost lines of any multiplet. The other reason to start with the largest coupling constants is that values of $J^{*}$ = J/(2n+1) (where $n\in N^{*}$ ) also produces a multiplet structure with no marginal signal (see the extremum at $J^{*}$ = J/3 in Fig. 3), making it unreliable for using symmetry as a criterion for the determination of values smaller than one-third of the largest coupling of the multiplet. This problem becomes irrelevant when using a recursive process where the result of the simplification of the largest coupling constants is used as the starting point of the next step. In order to limit the accumulation of artifacts, applying symmetry at each step or taking the best part of the sub-multiplets $S^{L}$ and $S^{R}$ is generally recommended.

An alternative to the measure of symmetry consists in testing the sum of the absolute values of the resulting multiplets. It should reach half the value of the starting multiplet when it is simplified. It requires only $M^{L}$ and $M^{R}$ (instead of both for the comparison using symmetry), but we used it solely when only one side was available, for example when analysing partially overlapping multiplets (see Sect. 1.8).

Having a good starting multiplet is also important and requires the spectrum to present a reasonable baseline, and the identification and subtraction of solvent and other artifact peaks by the processing software prior to the deconvolution process increase the chances of success of multiplet deconvolution. On the other hand, random noise should only impact the confidence level of the identification of each coupling.

### 1.4 Multiplet line shape

**Figure 4:**
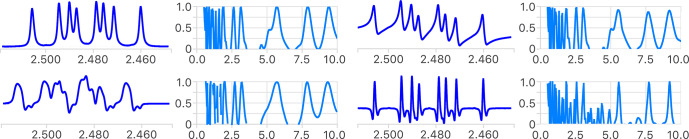
Comparison of the first step of the analysis of the reference multiplet before (top left) and after introduction of a phase error (top right), a distorted line shape (bottom left) and using its second derivative (bottom right). The measured coupling constants are the same in all cases (not shown), as the position of extrema in the four error functions shows. The narrower extrema of the error functions in the latter indicate a higher potential to identify small coupling constants. See Fig. S1 for a more detailed analysis.

An interesting property of multiplet deconvolution is to make no assumption about the underlying signals' line shape. It can be any mixed Lorentz/Gauss function, transformed by any type of apodization or line-shape deconvolution. Multiplet deconvolution only needs a pattern, of any shape, to be equally split. Figures 4 and S1 in the Supplement illustrate this point for a classical phase distortion, a distorted line shape, and the results of a resolution enhancement replacing the multiplet with its second derivative (Wahab et al., 2018). This being said, the above-mentioned symmetrization procedure applied at each step of the simplification process is generally beneficial (compare Figs. S2 and S3 in the Supplement). It averages out noise, artifacts and asymmetrical coupling structures caused by mild second-order effects (roof effects), but it usually does not work properly when signals do not have a pure absorption line shape (i.e. when the phase is incorrect) or when the $B_{0}$ inhomogeneity produces a severely asymmetrical line shape.

### 1.5 Spin system degeneracy

When spin systems are n -fold degenerated, the convolution with $M(J^{*})$ is simply applied n times before measuring the degree of simplification. A trivial example of quartet structure is shown in Fig. S4 in the Supplement. A user-controlled specification of degeneracy simplifies the structure in one step and avoids the possible errors of the automatic identification which takes as *degenerate* the coupling constants differing by less than 0.5 Hz .

**Figure 5:**
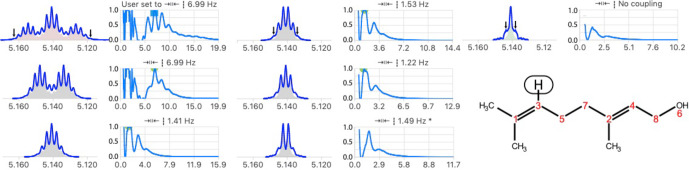
Example of automatic analysis failing to identify a triplet structure after six steps of simplification. Because the outermost lines (see arrows) were too small, it identified a problem because the reconstructed multiplet (triple quintet filled in red) was not perfectly matching (the scalar product was < 0.99) the starting multiplet (in blue). The post-analysis procedure found a satisfactory match (not shown) for a triplet (7.0 Hz ) caused by methylene (C5) of heptet ( ${}^{4}J$ = 1.3 Hz ) caused by the coupling with two methyl groups bound to C1 of geraniol.

Another difficulty arises, in highly degenerate systems, when the automatic analysis misses the outermost signals (see Fig. 5).

A post-processing procedure using multiplet simulations (see Sect. 2.3) to test whether higher levels of degeneracy match better the starting multiplet turned out to be useful, including in the case illustrated in Fig. 5.

### 1.6 Coupling partner with S>1/2

**Figure 6:**
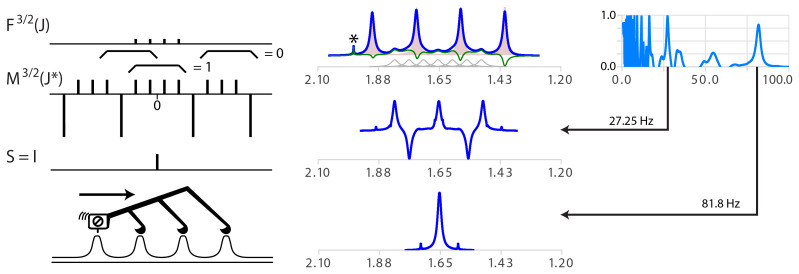
(Top right) Model multiplet structure for coupling with S=3/2 partners. (Middle right) Simplifying series of δ functions $M^{3/2}(J^{*})$ . The braces show examples of integration regions and illustrate that it is zero for all positions except the central one. The side-to-side process is quite similar to the case of S=1/2 except that subtraction of the cursor position has to be made to the n-1 positions located $J^{*}$ further in the data array. (Centre) Analysis of the proton spectrum of ${\mathrm{BH}}_{4}^{+}$ . Specifying the spin of the partner as 3/2 replaces the wrong assignment dd ( J = 163.6, 81.6 Hz , not shown) with the correct 1:1:1:1 quartet with J = 81.6 Hz (bottom centre). The extremum of the quality function (right) for $J^{*}=J/3$ = 27.25 is due to a non-simplified but symmetric structure (bottom centre). The presence of the minor multiplet for the ${}^{10}B$ isotope (20 % natural abundance, S=3 , seven transitions) with a γ one-third that of ${}^{11}B$ is highlighted in grey in the top subplot. Note that the artifact (probably traces of acetone highlighted by a star symbol) was scaled down by symmetrization.

When compounds contain deuterium ( S = 1, 1:1:1 structure), boron ( ${}^{11}B$ , S = 3/2 , 1:1:1:1 structures) and other atoms with S>1/2 isotopes, the simplification of the multiplet structure with n equal-amplitude δ function is necessary when quadrupolar relaxation does not effectively hide the coupling structure. The general inverse function $M(J^{*})$ , valid for S≥1/2 , consists of a core of nδ functions with unit values producing the main singlet. It is flanked at both sides by a repeat of blocks made of one negative δ function with intensity equal to -(n-1) and n-1 lines with unit intensities. The function $M^{3/2}(J^{*})$ is shown in Fig. 6.

The convolution product of $F^{3/2}(J)$ and $M^{3/2}(J^{*}=J)$ is indeed I because the sum of any set of four consecutive δ functions $\left(\sum_{i=m}^{m+n}\delta_{i}\right)$ is equal to zero for all positions except the middle one. The side-to-side recursive functions $M^{L,\;S>1/2}(J^{*})$ and $M^{R,\;S>1/2}(J^{*})$ also exist (see $M^{L}$ at the bottom left of Fig. 6).

For a given coupling constant, the density of δ functions in the simplifying function is the same as for spin 1/2 , but the fact that the average of the intensities of the M functions increases with spin order and that these multiplets are more extended should generally make the simplification of complex multiplets more prone to artifacts.

The analysis of the main 1:1:1:1 quartet of the proton of borohydride produced by the coupling with ${}^{11}B$ is straightforward (see Fig. 6). The process is not disturbed by the presence of the signal of the 20 % abundance of the ${}^{10}B$ isotopologue. Note that a refinement of the parameters involving a fit of the experimental spectrum with spectral simulation taking into account the presence of isotopologues could provide values for the isotopic shift and the line widths of both multiplets.

The analysis of a system including the deuterium atoms of the ${\mathrm{CHD}}_{2}$ residual signal of DMSO-D6 is shown in Fig. S5 in the Supplement.

### 1.7 Mild second-order effects

**Figure 7:**
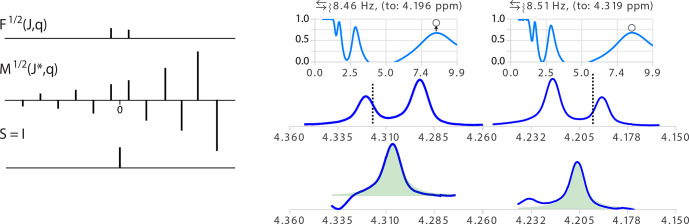
(Left) Illustration of the model function of a doublet with non-equal amplitude $F^{1/2}(J,\theta )$ and its inverse function $M^{1/2}(J^{*},\theta )$ . The amplitude ratio of the absolute values of two consecutive δ in M is the inverse of the ratio in $F^{1/2}(J,\theta )$ . (Right) Analysis of two multiplet structures of an AB spin system (300 MHz spectrum of a taxol sample). Δδ/J = 6.6, 2θ = 8.6 deg. and the ratio of signal amplitudes r = 0.74. The error in the determination of partner chemical shift was about 0.01 ppm . The open circles in the quality functions indicate the level of the measure of the quality function after optimization of θ .

Because second-order effects are often observed in otherwise well-behaved multiplets (i.e. when only the peak intensities are uneven with no significant shift or additional second-order transitions), we introduced, in this work, the function deconvoluting doublets with non-equal amplitudes. Mild second-order effects usually do not impair the extraction of coupling constants, especially when symmetrizing multiplet structures, but the validation of the results matching simulation with the experimental multiplet (see Sect. 2.3) may fail because of the mismatch of the peak amplitudes. Accounting for the roof effect restores high confidence in the results of the analysis. The simplification function $M^{1/2}(J^{*},\theta )$ (see Fig. 7) includes the strength of the coupling as a parameter expressed a $\theta ={\text{tan}}^{-1}(J/\Delta \delta )$ where Δδ is the difference in the chemical shift of the two coupling partners expressed in Hertz. It produces the identity function when taking the convolution product with the second-order doublet $F^{1/2}(J,\theta )$ where the ratio of peak amplitudes is

1 $$r=\frac{1-\mathrm{sin}(\theta )}{1+\mathrm{sin}(\theta )}.$$

Note that the stronger the coupling, the more artifacts will be amplified on one side of the resulting multiplet. In order to avoid having two independent variables to adjust, θ can be optimized only for values of $J^{*}$ corresponding to an extremum of the values obtained for θ=0 .

Besides the fact that taking into account the roof effects increases the level of confidence that any measure splitting is correct, exploiting them provides, when it can be measured with sufficient precision, the chemical shift of the relevant coupling partner (Stan Sykora​​​​​​​, personal communication, 2008). This information normally requires a 2D COSY spectrum, but extracting this information for the 1D spectrum has the additional advantage of identifying which of the couplings observed in a given multiplet corresponds to the designated coupling partner. In other words, instead of only qualitatively pointing to the side of the spectrum where one should look for the coupling partner of a signal showing roof effects, one can actually point to the chemical shift of the partner according to

2 $$\delta_{\text{partner}}=\delta_{\text{ref.}}+\frac{J}{\text{tan}(\theta )}.$$

The determination of the coupling partner position is expected to provide useful values for a relatively narrow range of second-order effects (say 3 < Δδ/J < 20). In the example of Fig. 7, taken from the ${}^{1}H$ spectrum of a sample of taxol (Peng et al., 1997), Δδ/J = 6.6. Indeed, the second-order effect should not be too strong to prevent the minor line of the doublet from being too small to be measured accurately. Similarly, if the effect is too weak, the difference of intensity of the two lines of the doublet becomes too small to be significant and generate large errors because 1/tan(θ) becomes quite large when θ is small (see the example in Fig. S6 in the Supplement). Taking into account the error in the partner position by standard error propagation calculation is therefore essential to avoid misinterpretation. Obviously, the chances of success of simply picking the nearest multiplet about the expected chemical shift as the true partner depends on the complexity of the spectra.

### 1.8 Partially overlapping multiplets

**Figure 8:**
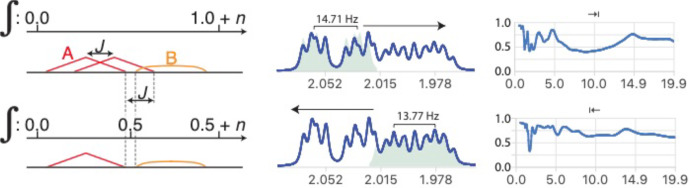
Analysis of multiplet A in the presence of partially overlapping multiplet B with relative integrals of 1 and n , respectively. When deconvolution is successful, the left-to-right deconvolution process eliminates the second occurrence of sub-multiplet A which effectively eliminates overlap (bottom left). (Right) Example of separation of two slightly overlapping multiplet structures from a sample of artemicinin dissolved in ${\mathrm{CDCl}}_{3}$ . The coupling constants J = 14.71, 4.77, 2.89 and 13.77, 6.15, 3.91 and 2.93 Hz were extracted from the left and right multiplets, respectively, and used to reconstruct the matching green multiplets. Note the slight mismatch due to second-order effects which could be ignored in this analysis. See Fig. S7 in the Supplement for the detailed simplification.

The side-to-side deconvolution algorithm allows us to deal with multiplets that are partially superposed. Figure 8 shows that signal overlap over a distance smaller than the largest coupling present in the multiplet can be analysed successfully.

When attempting to simplify the left multiplet, the deconvolution process should be run from left to right and the deconvolution process stopped at the position where the integral reaches 1/2 in the reconstructed multiplet.

When testing different values of $J^{*}$ , the measure of the success of the simplification cannot be applied to the whole spectral region because the segment where the sub-multiplet subtraction occurs may be occupied by multiplet B. However, successful deconvolution produces a minimal and predictable integral when $J^{*}=J$ . This multiplet separation method requires us to know the integral of the analysed multiplet relative to the total integral of the region of interest. This is usually not problematic as integration is generally straightforward. An example of analysis resulting in a sub-multiplet separation is shown in the right part of Figs. 8 and S7.

Following the analysis of A, multiplet B can be analysed independently (from right to left) only if its largest coupling is also large enough, as in the example on the right of Fig. 8. Otherwise, a recursive process analysing and subtracting multiplet structures sequentially is necessary. This would make the quality of the subtraction of each multiplet high enough to leave no significant residual signals on the rest, but allows us, in principle, to deal with more than two overlapping multiplets.

For more severely overlapping structures, the methodology developed to separate 2D multiplets (Jeannerat and Bodenhausen, 1996) could be adapted to the processing of 1D spectra.

## 2 Implementation

### 2.1 Deconvolution parameters

As mentioned above, the simplification of structure is a recursive process where the noise and artifacts tend to increase along the course of the analysis and depend on the quality of the multiplet (complexity, baseline distortion, presence of spurious peaks, etc.).

In order to increase the robustness of multiplet deconvolution, different sets of parameters driving the deconvolution process are tested and their results validated (see next paragraph). These parameters are the threshold for considering a deconvolution to be successful, the threshold for considering the recursive process to be completed, the range of values tested for the roof effect, whether symmetry is applied before starting the analysis, whether symmetry is applied after each simplification, whether the baseline offset is corrected, whether the crude spectrum is used instead of the GSD-based synthetic spectrum (Cobas et al., 2008; Schoenberger et al., 2016), etc.

**Figure 9:**
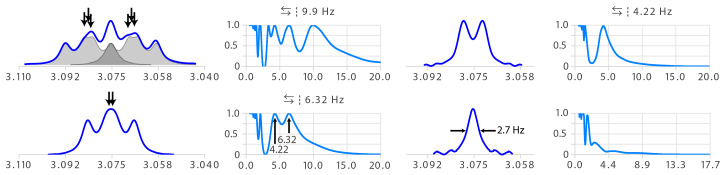
Example of a *ddd* structure analysed straightforwardly by multiplet deconvolution. The symbol indicates that symmetrization was applied before deconvolution. The symbol indicates that the results of the deconvolution running from both sides were added (instead of taking the best part of both of each or only the result of one direction).

### 2.2 Limit of deconvolution analysis

Obviously, the recursive simplification stops when the result is a singlet. However, the ability of multiplet deconvolution to find unresolved coupling means that simply testing the presence of only one extremum in the result of a simplification step could miss unresolved coupling constants (see Sect. 3.2). Testing values down to 1 Hz seems to be a good compromise for standard 1D ${}^{1}H$ spectra. Note that sub-linewidth coupling constants should be used with great care and only to facilitate the process of a structure elucidation process. They should not be used as the sole argument for any key interpretation. Indeed, the behaviour of the deconvolution has not been studied sufficiently to provide a general and safe limit as to how far out it can be carried. A rigorous analysis of unresolved structures would involve a process taking, among other elements, the line shape found in other multiplets into account. When a candidate structure is available, predictable long-range coupling constants should be considered. Depending on the priority of the application, one may favour safety over ambition and decide to ignore any unresolved structure in a “fully automated” mode. Pushing the limit of multiplet analysis could be a user-triggered operation providing visualization tools to assess the validity of the results and correct them if necessary.

### 2.3 Validation and post-processing

The validation and ranking of the results of the different methods consist in the measure of the similarity of the starting multiplet to a reconstructed multiplet build using the extracted data. The latter are obtained by starting with the final singlet shape and reintroducing the measured coupling constants including, when relevant, the tilt caused by the second-order effect, and testing the similarity with the initial multiplet. In this work, the starting line shapes were synthetic Lorentzian but allowing more degrees of freedom (i.e. using Voigt, generalized Lorentzian (Schoenberger et al., 2016), etc., line shapes) should improve the match with the shape obtained at the end of the deconvolution.

Finally, a refinement of all parameters, signal intensity, chemical shift, coupling constants, but also roof effect, signal phase, baseline level, and line-shape parameters, could be used to further improve the quality of the validation and take into account context-dependent information such as the degeneracy of the coupling constants.

## 3 Results and discussion

Multiplet deconvolution has been applied to numerous test spectra. Many multiplet structures which could not be analysed using the GSD peak-based multiplet analysis (reported as “ m ”) were correctly deciphered. It also has a lower occurrence of false positives – the situation where coupling constants are automatically extracted but turn out to be incorrect – thanks to the strict validation process. We shall only present here two typical examples where multiplet deconvolution succeeds while manual analysis would be difficult.

### 3.1 Human-unfriendly multiplets

Figure 9 shows a typical example of a multiplet that causes difficulties for human analysis. The partial overlap of three transitions (see arrows) makes the structure difficult to interpret. The destiny of such a structure is to be called the bad name “ m ” for a lack of courage and proper tool to identify it. Multiplet deconvolution straightforwardly identifies the largest coupling as 9.9 Hz . Similarly, the seeming triplet of the second step (bottom left of Fig. 9) would have been assigned to a t with two equal coupling constants of 5.27 Hz – possibly causing confusion if nothing explains this apparent degeneracy. Here again, deconvolution has no difficulties in resolving overlap and identifies a double doublet with 6.32 and 4.22 Hz – an almost 2 Hz difference for a final line width of 2.7 Hz .

An example of successful analysis of a “*dqdd*” multiplet structure which was too complex to be analysed manually is shown in Fig. S8 in the Supplement. The six coupling interactions, among which three were degenerate, produced 32 lines with no visible splitting patterns.

**Figure 10:**
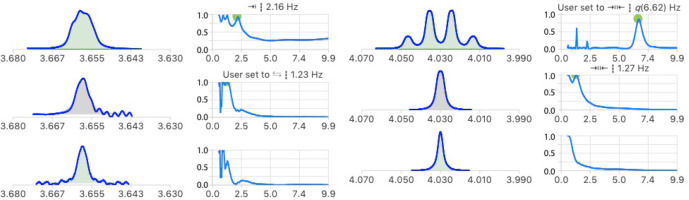
Extraction of unresolved coupling constants of the proton signals of C4 (left) and C5 (right) of a 6-deoxy-pyranose unit. The symbol indicates that symmetrization was not applied and deconvolution run from left to right.

### 3.2 Unresolved couplings

In carbohydrate chemistry, geminal coupling constants are often too small to be resolved, making it difficult to assign their signals. The analysis of the multiplet structures of Fig. 10 identified a common coupling of 1.2 Hz which increases the confidence in their suspected geminal relationship. In the signal at 3.655 ppm , neither the 2.2 nor 1.2 Hz were resolved in the starting multiplet, providing a good example of the potential of multiplet deconvolution to access useful coupling constants.

## 4 Conclusions

The recursive analysis of NMR spectra by multiplet deconvolution demonstrated its ability to extract coupling constants in a robust and fully automated manner. The results are validated when simulations based on the extracted data match the experimental multiplet structures. Additional features including the analysis of structures produced by coupling partners with S>1/2 and of regions presenting signal overlap are proposed with the option of using a graphical interface providing a full user control on the stepwise analysis of multiplet structures.

By reducing the number of unsuccessfully analysed multiplets, multiplet deconvolution increases the information content of NMR spectra at a much lower cost than increasing the field strength of NMR spectrometers. Combined with other efforts aiming at automatizing the analysis of NMR spectra, these methods can significantly increase the quantity and quality of NMR data available to the community. However, this will only occur if researchers make their NMR spectra and extracted data available as supplementary data deposited in public databases instead of providing only crude images of spectra and other non-computer-readable information.

## Supplement

10.5194/mr-2-545-2021-supplement The supplement related to this article is available online at: https://doi.org/10.5194/mr-2-545-2021-supplement.

## Data Availability

The software presented here is the property of Mestrelab, but the code for a basic recursive deconvolution of a first-order multiplet with spin partners is copyright free and available as JavaScript node modules on https://doi.org/10.5281/zenodo.4973506 (Jeannerat and Patiny, 2021a). The JCAMP-DX files of the spectra presented in this paper are available on Zenodo (https://doi.org/10.5281/zenodo.4616665, Jeannerat, 2021). They can be visualized with the open-source NMR displayer (also known as NMRium) accessible from https://doi.org/10.5281/zenodo.4973419 (Jeannerat and Patiny, 2021b) and analysed by the basic implementation of deconvolution by clicking on the Ranges Picking button and selecting a multiplet region with the mouse while pressing the shift key.
