# Supplementary material for: Application of multiplet structure deconvolution to extract scalar coupling constants from 1D nuclear magnetic resonance spectra
Source: Magn Reson (Gott). 2021 Jul 6;2(2):545–55. doi: 10.5194/mr-2-545-2021 (PMC10539725; doi:10.5194/mr-2-545-2021)
Supplement: The supplement related to this article is available online at: https://doi.org/10.5194/mr-2-545-2021-supplement. [file mr-2-545-supplement.pdf]

Supplement of Magn. Reson., 2, 545–555, 2021
https://doi.org/10.5194/mr-2-545-2021-supplement
© Author(s) 2021. CC BY 4.0 License.

Open Access
MAGNETIC RESONANCE

Supplement of

Application of multiplet structure deconvolution to extract scalar coupling constants from 1D nuclear magnetic resonance spectra

Damien Jeannerat and Carlos Cobas

Correspondence to: Damien Jeannerat (damien.jeannerat@protonmail.com)

The copyright of individual parts of the supplement might differ from the article licence.

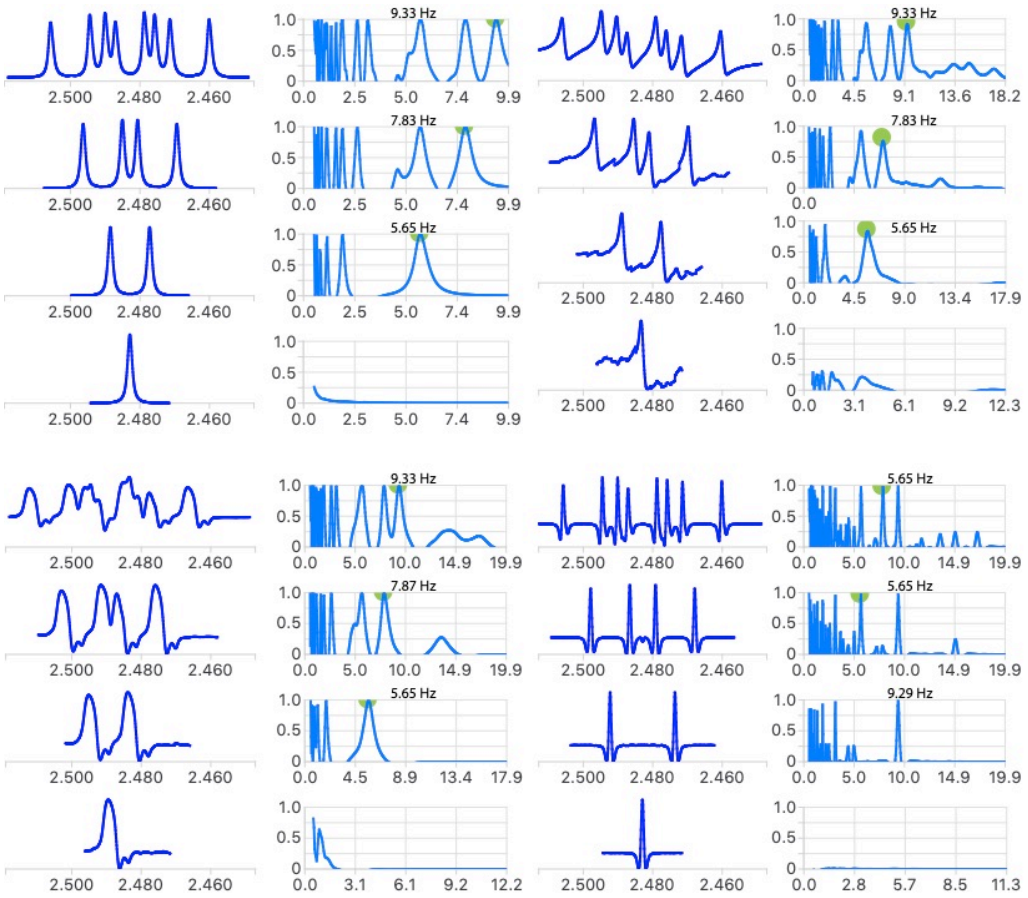

Figure S1: Comparison of the analysis of a multiplet structure before (top left) and after introduction of a phase error (top right), a distorted line-shape (bottom left) and the application of resolution-enhancement method using its second derivative (bottom right). The measured coupling constants are the same in all cases. The narrower extrema of the error functions in the latter indicate a higher potential to identify small coupling constants. Figure 4 illustrates only the first step of the analysis.

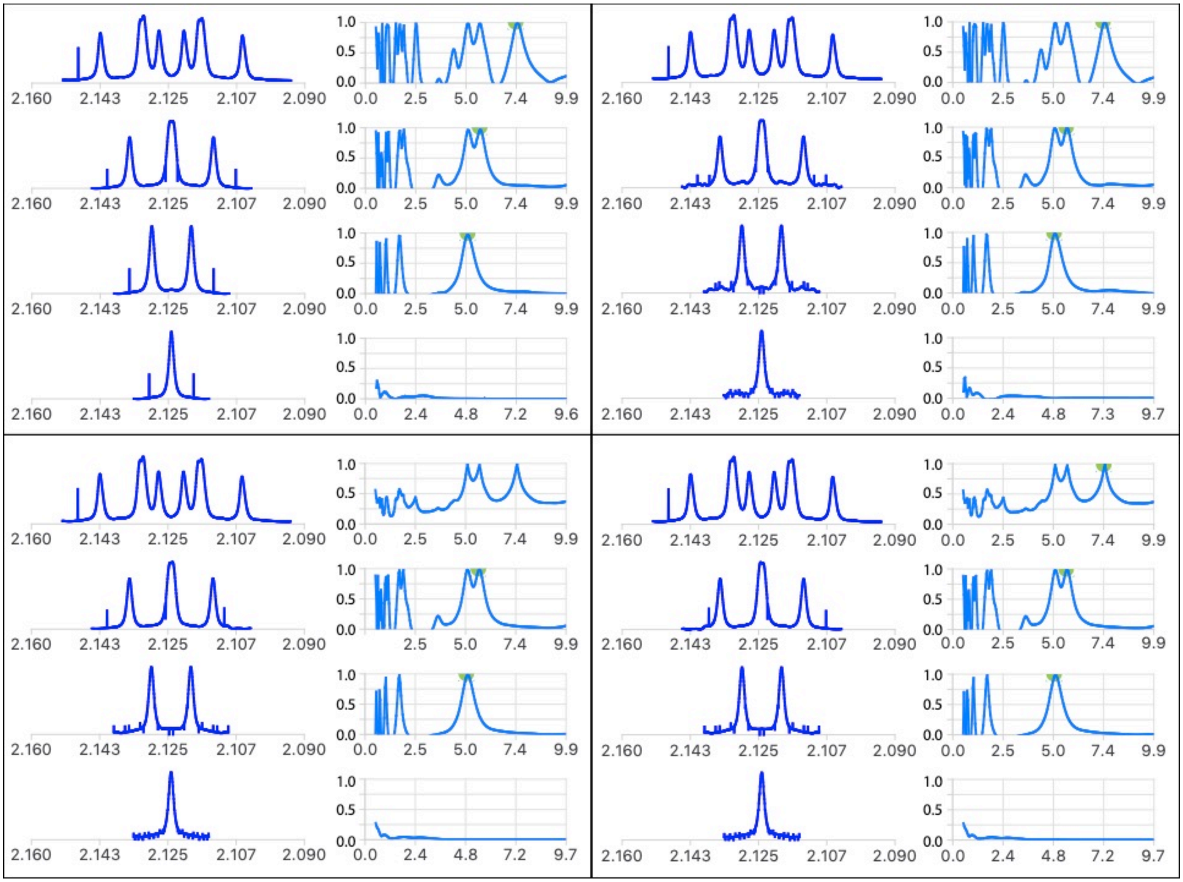

Figure S2: Comparison of the impact of a spike added to the starting multiplet when using different pre-deconvolution processing applying symmetrization at each step of the simplification. Results obtained using (top left) the sum of S^L and S^R, (top right) the best part of S^L and S^R (bottom left) only S^L and (bottom right) only S^R. See Fig. S3 for the analysis without symmetrization.

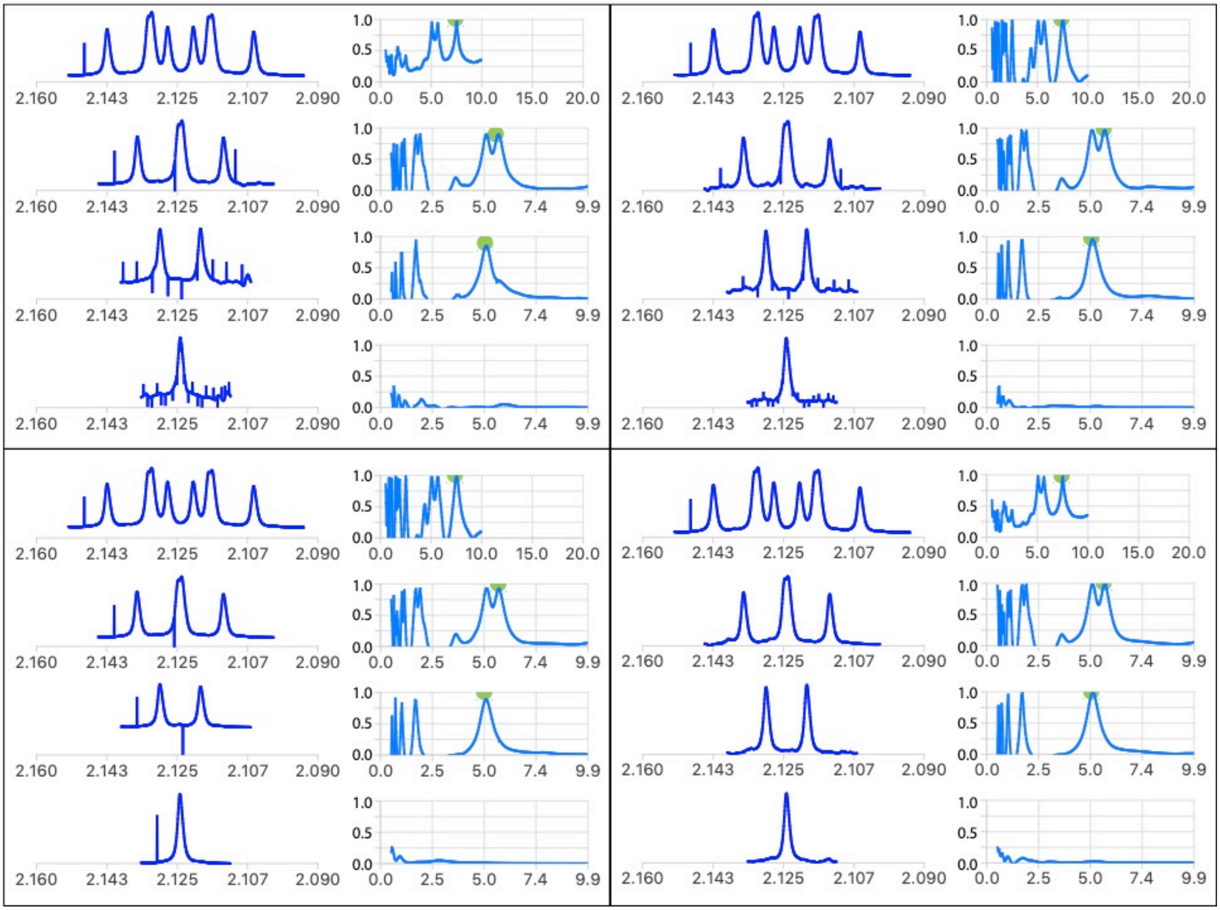

Figure S3: Comparison of the impact of a spike added to the starting multiplet when using different pre-deconvolution processing without applying symmetrization at each step of the simplification. Results obtained using (top left) the sum of S^L and S^R, (top right) the best part of S^L and S^R (bottom left) only S^L and (bottom right) only S^R. See Fig. S2 for the analysis with symmetrization.

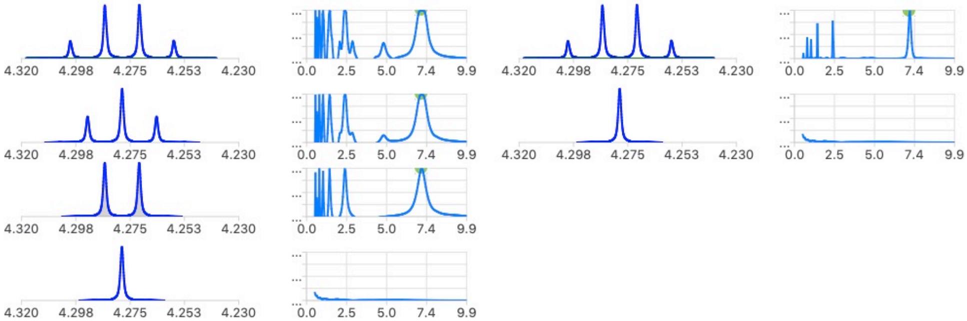

Figure S4: Comparison of the standard analysis resulting to a *ddd* ($J =$ 7.15, 7.15, 7.15 Hz) with the one obtained when the quartet degeneracy of the multiplet was set by the user to be a quartet *q* ($J = 7.15$ Hz).

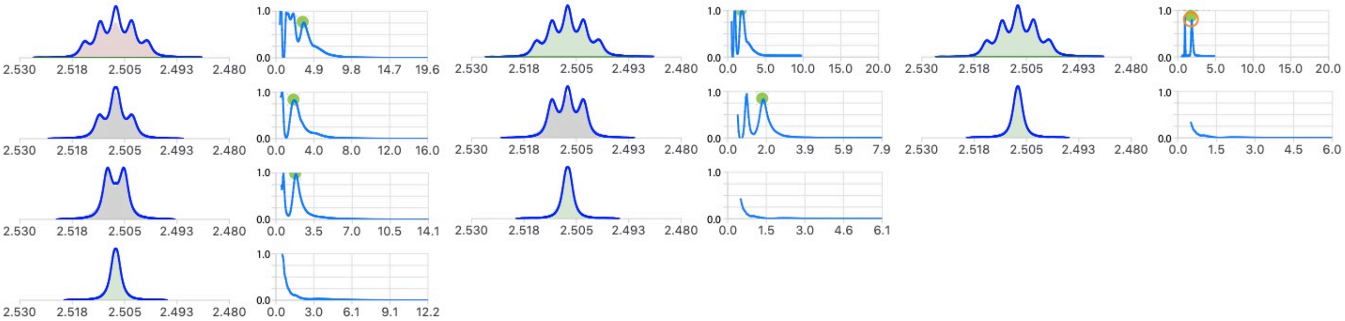

Figure S5: Analysis of the residual signal of the proton of the CHD_2 of DMSO-D6. (left) Initial analysis wrongly assuming coupling partners with spin 1/2 identifies a *ddd* (*J* = 3.57, 1.95, 1.86 Hz) but the validation rejected it because of an insufficiently good match (scalar product < 0.99) of the reconstructed structure (filled in red) with the starting structure (see the missing surface relative to the reconstructed structure in the central peak). After setting the partner spin to 1, the analysis is correct (centre) with $J = 1.86$ and 1.81 Hz and specifying the degeneracy makes in a single-step process (right) returning $J = 1.86$ Hz.

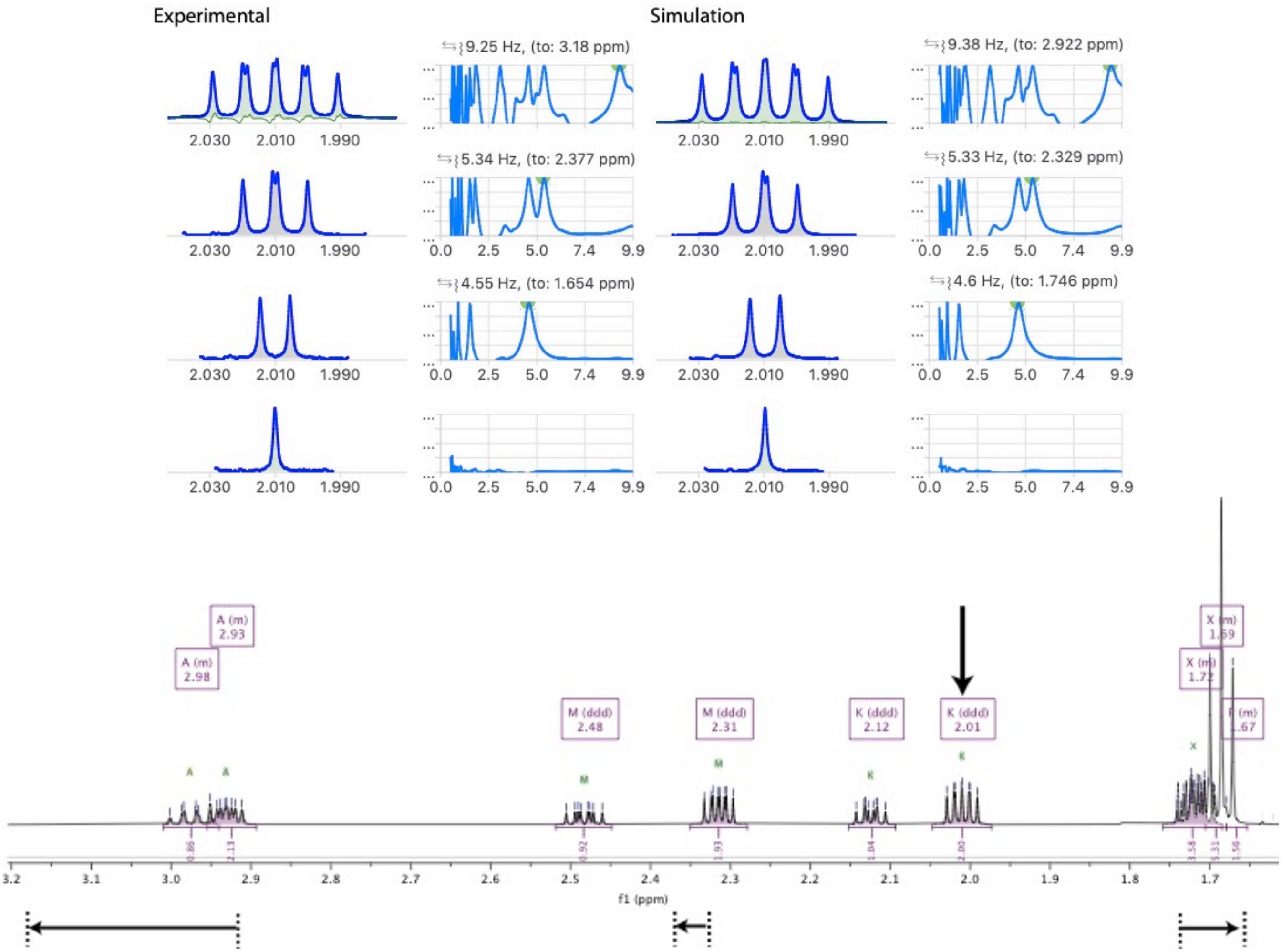

Figure S6: Illustration of the attempt to exploit the second-order effects to determine the partner position of a proton (*ddd* multiplet at 2.01 ppm) of a cyclopropane derivative. The coupling constants were J = 9.38, 5.34 and 4.55 Hz for partners at 2.93, 2.31 and 1.72 ppm respectively. (left) The partner chemical shifts calculated from the slope of the experimental multiplet were 3.18 (+0.25), 2.377 (+0.066) and 1.654 (−0.056) ppm respectively with $\Delta\delta/J$ = 59, 33 and −39 respectively, 2θ = 0.97, 1.70 and −1.44 deg. and r = 0.97, 0.94 and 1.05. The error in chemical shifts was less than 0.1 ppm for $\Delta\delta/J < 50$. (right) Results of the analysis based on simulations. The partner chemical shifts calculated from the slope of the simulated multiplet structure were 2.922 (+0.008), 2.329 (−0.019) and 1.746 (−0.026) ppm. (bottom) Proton spectrum of the reference multiplet (vertical arrow) and its coupling partners. The horizontal arrows run from the predicted chemical shifts originating from the simulated spectrum to the ones determined from the experimental data. The ^{1}H-Larmor frequency was 600 MHz.

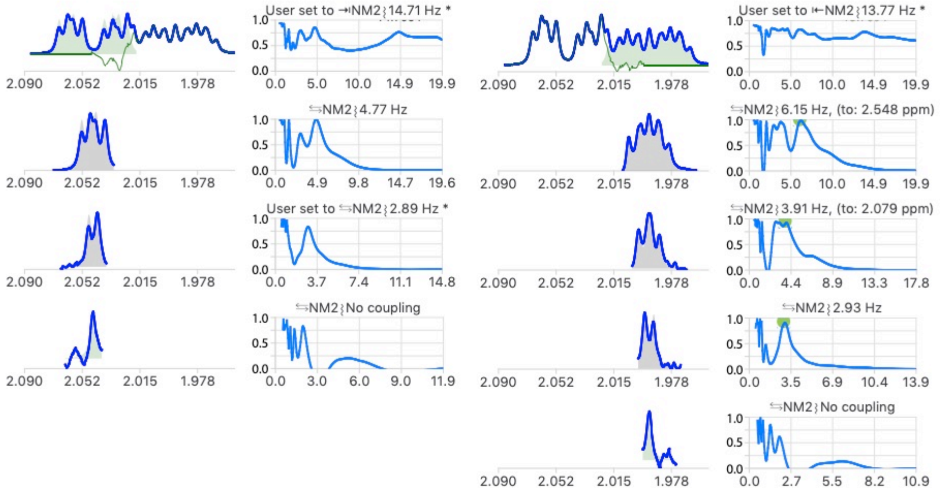

Figure S7: Details of Fig 8.

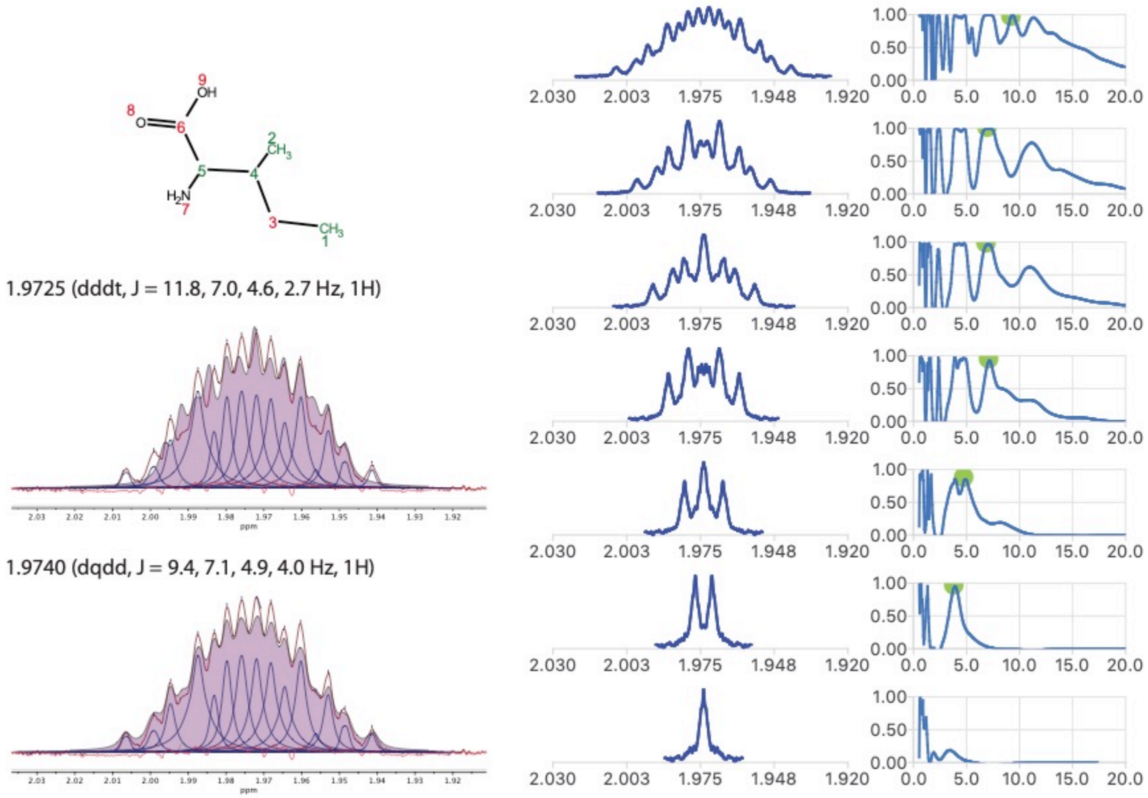

Figure S8: (top) Comparison of the analysis of the standard peak-based method (middle left) and multiplet deconvolution (bottom left and right) of the proton bound to the C4 carbon of isoleucine. The coupling constants resulting to the correct *dqdd* structures are 9.31, 7.05, 6.96, 7.2, 4.85 and 3.92 Hz were compatible with the values found in the multiplet patterns of the coupling partners.
